# Atherogenic index of plasma and cardiovascular high-risk status in the ChinaHEART luohe cohort: multivariable association modeling with nonlinear dose-response and effect heterogeneity

**DOI:** 10.3389/fendo.2026.1798735

**Published:** 2026-04-01

**Authors:** Jing Bai, Jirui Cai, Zhiwei Huang, Jin Wang, Bing He, Li Guo, Li Wu, Dongliang Liu, Guirang Zhao, Qiaotao Xie, Haoran Wang

**Affiliations:** 1 Luohe Central Hospital, Luohe Medical College, Luohe, China; 2 Henan University College of Medicine, Henan University, Kaifeng, China; 3 Luohe Center for Disease Control and Prevention, Luohe, China

**Keywords:** atherogenic dyslipidemia, atherogenic index of plasma, cardiovascular disease risk, ChinaHEART, community screening, WHO CVD risk charts

## Abstract

**Background:**

Community screening programs increasingly use World Health Organization (WHO) cardiovascular disease (CVD) risk charts to identify individuals at high predicted 10-year risk. The atherogenic index of plasma (AIP), derived from triglycerides (TG) and high-density lipoprotein cholesterol (HDL-C), may capture atherogenic dyslipidemia and support pragmatic risk stratification.

**Methods:**

We conducted a cross-sectional analysis of baseline data from the China Health Evaluation And risk Reduction through nationwide Teamwork (ChinaHEART) community screening program in Luohe, China. Among 6,860 screened participants, 6,702 with complete data for AIP computation, WHO risk classification, and prespecified covariates were included. The outcome was the WHO CVD risk chart-defined predicted 10-year CVD high-risk category (high risk: ≥20%), rather than adjudicated or incident CVD events. AIP was calculated as log10(TG [mmol/L]/HDL-C [mmol/L]) and modeled as both a continuous and categorical exposure; spline models tested nonlinearity, and ROC analyses evaluated discrimination and derived a Youden-index cutoff. In addition, we performed an explainable machine-learning pipeline for CVD high-risk prediction using LASSO logistic regression for feature selection (AIP forced-in), followed by a random forest classifier and SHAP-based interpretation.

**Results:**

Of 6,860 screened participants, 6,702 were included in the analytic sample (median age 58 years; 38% men). The WHO CVD risk chart-defined predicted 10-year CVD high-risk category was present in 1,440 (21%) participants and was more frequent in the high-AIP group than in the low-AIP group. Higher AIP was associated with higher odds of CVD high-risk status. Restricted cubic splines supported a non-linear association. Discrimination was modest for AIP alone (AUC 0.557) and improved in adjusted models (AUC 0.650). In the machine-learning pipeline (LASSO + random forest), the random forest model achieved an AUC of 0.792, and SHAP analyses ranked LDL-C and history of hypertension as the strongest contributors, with AIP remaining among the top predictive features.

**Conclusion:**

In this community-based ChinaHEART population, higher AIP was non-linearly associated with the WHO CVD risk chart-defined predicted 10-year CVD high-risk category. Although AIP alone had limited discrimination, it may serve as a simple adjunct marker to triage individuals for intensified risk assessment in primary-care screening settings.

## Introduction

Cardiovascular disease (CVD) remains a major cause of premature death and disability, and prevention depends on identifying individuals at high absolute risk who are likely to benefit most from intensified risk-factor management (1, 2). In community settings—especially where clinical resources are constrained—risk stratification frequently relies on scalable, standardized tools such as the World Health Organization (WHO) CVD risk charts to estimate 10-year CVD risk and guide prevention intensity (3–6).

Atherogenic dyslipidemia, typically characterized by higher triglycerides (TG) and lower high-density lipoprotein cholesterol (HDL-C), is closely linked to insulin resistance, central adiposity, and systemic inflammation (7–11). This lipid pattern is common in middle-aged and older adults and may contribute to higher absolute CVD risk. However, interpreting TG and HDL-C in isolation may miss their combined atherogenic implication. The atherogenic index of plasma (AIP), computed from TG and HDL-C, was proposed as a simple composite marker that summarizes this dyslipidemic phenotype (12, 13). Because TG and HDL-C are routinely measured in screening programs, AIP is attractive as a pragmatic marker that can be calculated without additional costs.

Despite growing interest, it remains clinically relevant to clarify whether AIP is associated with WHO CVD risk chart-defined CVD high-risk status in community-dwelling adults, and whether the AIP-risk relationship is linear across the entire AIP distribution (14–20). Understanding these patterns can inform whether AIP could be used as a low-cost adjunct to prioritize individuals for more comprehensive assessment, counseling, and preventive intervention.

Therefore, using baseline data from the ChinaHEART community screening program in Luohe, central China, we investigated the association between AIP and WHO-defined predicted CVD high-risk status (3). We also (i) characterized potential non-linear dose-response patterns using restricted cubic splines, (ii) evaluated discriminatory performance and thresholds using ROC analyses with bootstrap-based confidence intervals, and (iii) explored heterogeneity through subgroup and interaction analyses.

## Methods

### Study design and population

This cross-sectional analysis used baseline data from the ChinaHEART screening program in Luohe, Henan, China (21, 22). Participants were invited through community outreach, and eligible residents who attended screening clinics during the study period were considered for inclusion. The screening targeted adults aged 35–75 years who were permanent residents of the study area (living in the community for ≥6 months in the preceding year) and who provided written informed consent.

A total of 6,860 residents attended baseline screening. For the present analysis, we included participants with available lipid measurements required to compute AIP, complete WHO CVD risk classification, and covariates specified in the main regression model (3). After excluding 158 participants with missing data in these variables, 6,702 participants were retained for the final analysis. The study protocol was approved by the ethics committee of Fuwai Hospital and filed with the local ethics committee of Luohe Central Hospital. All procedures complied with the Declaration of Helsinki (23), and reporting was prepared in accordance with the Strengthening the Reporting of Observational Studies in Epidemiology (STROBE) recommendations (24).

### Data collection and measurements

Trained healthcare staff conducted face-to-face interviews using standardized questionnaires to collect demographic characteristics, lifestyle behaviors (smoking and alcohol use), medical history (including hypertension, diabetes mellitus, and stroke), and medication use (including antihypertensive, lipid-lowering, and antidiabetic therapy).

Anthropometric measurements were performed according to a unified protocol. Waist circumference was measured at the midpoint between the lower margin of the last rib and the top of the iliac crest at the end of a gentle expiration. Blood pressure was measured with validated electronic sphygmomanometers after at least 5 minutes of seated rest, and two readings were averaged.

Fasting blood samples were obtained after an overnight fast, and lipid profiles (including TG and HDL-C) and fasting glucose were measured using standardized, regularly calibrated devices.

### Exposure definition: atherogenic index of plasma

AIP was calculated as log10(TG [mmol/L]/HDL-C [mmol/L]) (12). The original ROC/Youden-derived cutoff was retained for ROC reporting and sensitivity analysis, whereas a pragmatic rounded cutoff was used for the main descriptive, regression, and subgroup analyses. We evaluated AIP as (1) a continuous variable (per 1-unit increase) and (2) a categorical variable defined using the ROC-derived cutoff from the AIP-only ROC curve (Youden index). For practical use in clinical work and research, the ROC-derived cutoff was rounded, and the rounded value was used to classify participants into low vs high AIP groups for descriptive and categorical analyses.

### Outcome definition: CVD high-risk status

CVD high-risk status was defined using WHO CVD risk charts (3). Participants were classified as high risk if their predicted 10-year CVD risk was ≥20% and as non-high risk if it was <20%.

### Covariates and model specification

Covariates were selected *a priori* based on clinical relevance and the ChinaHEART screening context. Logistic regression models followed a stepwise adjustment strategy: Model 1 included AIP only; Model 2 adjusted for alcohol consumption and marital status; and Model 3 additionally adjusted for waist circumference, education, family history of stroke, antidiabetic drug therapy, antihypertensive drug therapy, and statin use.

### Statistical analysis

All statistical analyses were performed using R software (version 4.5.2). Continuous variables are presented as median (Q1 [first quartile], Q3 [third quartile]) and categorical variables as n (%). Between-group comparisons were conducted using the Mann-Whitney U test for continuous variables and Pearson’s chi-squared test for categorical variables. AIP was evaluated as both a continuous exposure and a categorical exposure, where the categorical cutoff was determined from the ROC curve using the Youden index and then rounded for practical application in routine clinical practice and research.

Binary logistic regression models were fitted to estimate the association between AIP and CVD high-risk status, using stepwise adjustment: Model 1 (crude), Model 2 (additionally adjusted for alcohol use and marital status), and Model 3 (further adjusted for waist circumference, education, family history of stroke, and medication use for diabetes, hypertension, and statins). Results are reported as odds ratios (ORs) with 95% confidence intervals (CIs). Dose-response relationships were explored using restricted cubic splines in logistic models (3 knots), with tests for overall and non-linear associations. ROC analyses were conducted for AIP-only and multivariable models; bootstrap resampling was used to obtain confidence intervals for AUCs and to estimate the 95% CI of the Youden-derived cutoff. A sensitivity subgroup analysis was additionally performed using the original cutoff.

### Machine-learning prediction and explainability analysis

To complement association modeling, we conducted an explainable machine-learning analysis to quantify multivariable predictive performance and feature importance. Candidate predictors were prespecified to reflect primarily modifiable/actionable risk factors available in community screening, so that feature-importance results could be interpreted as potential intervention targets. To minimize redundancy with AIP and reduce potential target leakage, triglycerides (a component of AIP) were not included in this pipeline; moreover, age and sex were excluded to keep the feature set focused on modifiable factors and to avoid over-dominance of non-modifiable predictors in feature-importance interpretation. We used LASSO logistic regression (10-fold cross-validation, AUC as the optimization metric; lambda.1se) for feature selection, forcing AIP into the penalized model by setting its penalty factor to zero. Variables retained by LASSO (plus AIP) were then used to train a random forest classifier (ntree=500) with a 70/30 train-test split. Predictive discrimination was evaluated by the AUC on the test set. Model interpretability was assessed using SHAP values estimated by Monte Carlo approximation (fastshap; nsim=30). In the SHAP summary plot, features are ordered by global importance, defined as the mean absolute SHAP value across participants. As a supplementary sensitivity analysis, we also refit the random forest + SHAP model after adding age and sex. This allowed us to reassess and compare the ranking of AIP among all included variables when age and sex were incorporated into the model.

## Results

### ROC analysis and determination of the AIP cutoff

**Figure 1:**
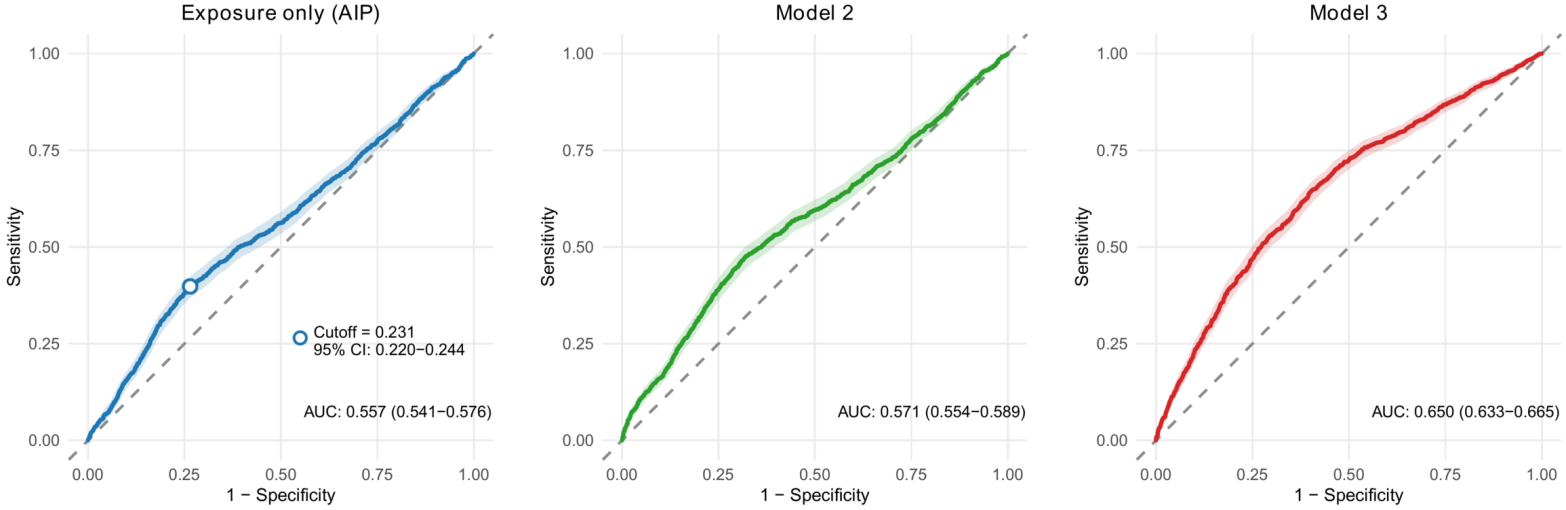
Receiver operating characteristic (ROC) curves for identifying WHO-defined CVD high-risk status. ROC curves compare discrimination of AIP alone and multivariable models (Model 2 and Model 3) for WHO CVD risk chart-defined predicted CVD high-risk status. AUCs and bootstrap confidence intervals are shown, and the Youden index provides the optimal AIP cutoff (0.231; 95% CI 0.220-0.244).

In ROC analysis, AIP alone showed modest discrimination for identifying WHO-chart-defined CVD high-risk status, with an AUC of 0.557 (95% CI 0.541-0.576). The optimal threshold derived from the Youden index was 0.231 (95% CI 0.220-0.244). Discrimination improved when AIP was combined with covariates, yielding AUCs of 0.571 for Model 2 and 0.650 for Model 3 (**Figure 1**).

For the main descriptive, regression, and subgroup analyses, a pragmatic rounded cutoff of 0.2 was used to define high AIP (AIP≥0.2) versus low AIP (AIP<0.2), whereas the original cutoff of 0.231 was used for additional sensitivity analysis, which showed that the overall direction of association remained similar (**Supplementary Figure 1**).

### Baseline characteristics by AIP group (cutoff = 0.2)

Among 6,702 participants, 5,225 (78%) were classified as low AIP and 1,477 (22%) as high AIP. Compared with the low AIP group, the high AIP group was slightly younger (median 57 vs 59 years, *P* = 0.024) and had a lower proportion of men (32% vs 39%, *P* < 0.001).

**Figure 2:**
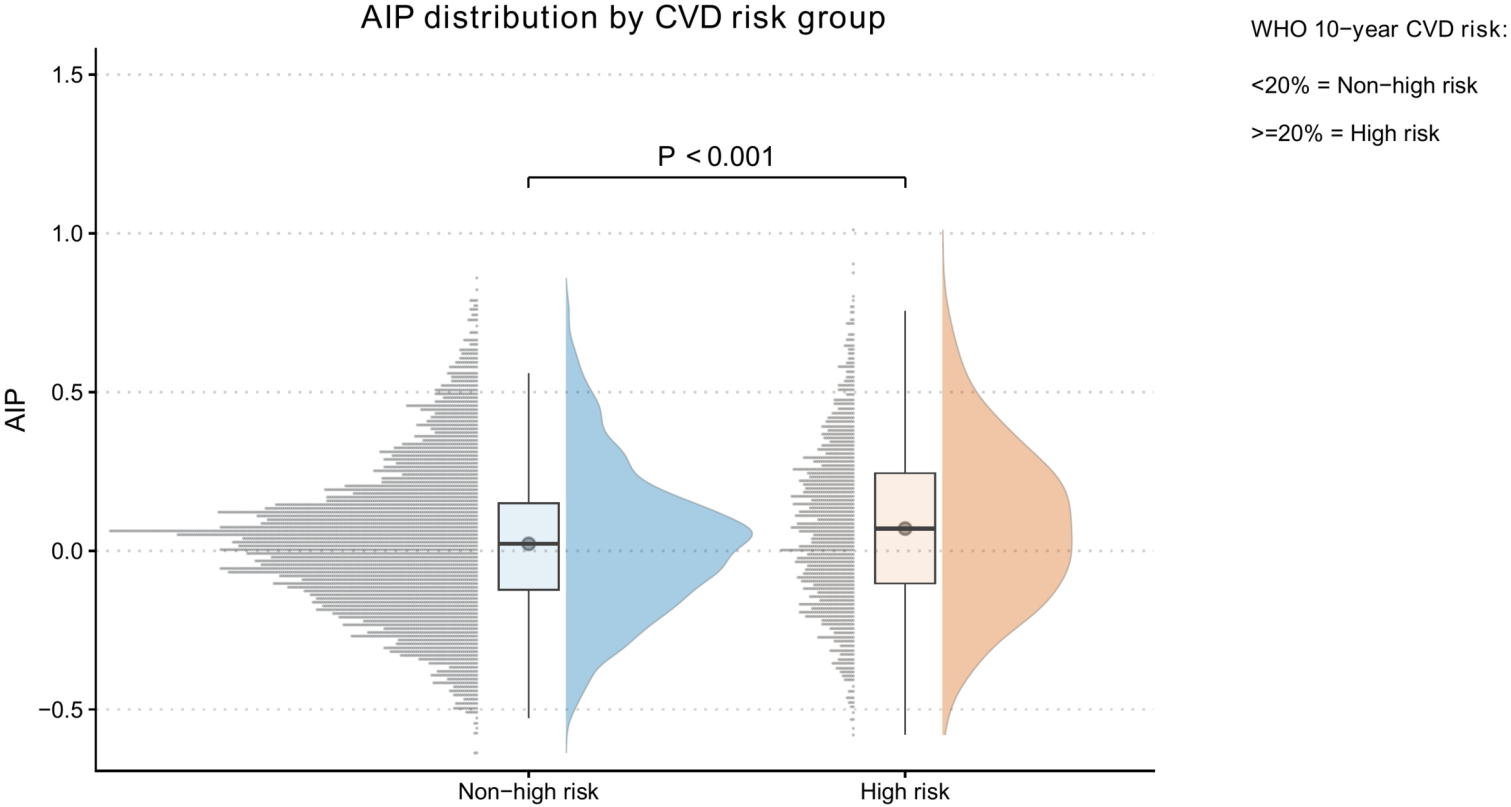
Raincloud plot of AIP distribution by WHO-defined CVD risk group. Raincloud plots display AIP distributions in non-high risk and high-risk groups defined by WHO 10-year CVD risk charts (≥20% vs <20%), combining density, boxplot, and raw-data visualization. Between-group difference was significant (*P* < 0.001).

However, the high AIP group exhibited more adverse cardiometabolic profiles, including higher waist circumference (0.90 vs 0.85 m), higher systolic/diastolic blood pressure (144/87 vs 136/82 mmHg), and higher fasting glucose (5.70 vs 5.40 mmol/L) (all *P* < 0.001). As expected, triglycerides were markedly higher and HDL-C lower in the high AIP group (TG 2.78 vs 1.37 mmol/L; HDL-C 1.21 vs 1.48 mmol/L; both *P* < 0.001). Notably, CVD high-risk status was more frequent in the high AIP group than in the low AIP group (31% vs 19%, *P* < 0.001) (**Figure 2** and **Table 1**).

**Table 1:** Baseline characteristics by AIP group (cutoff = 0.2).

Variable	Overall N = 6,702	Low AIP (<0.2) N = 5,225	High AIP (≥0.2) N = 1,477	*P*-value
Age (years)	58 (51, 66)	59 (51, 67)	57 (51, 65)	0.024
Sex (male)	2,518 (38%)	2,043 (39%)	475 (32%)	<0.001
Married	5,955 (89%)	4,658 (89%)	1,297 (88%)	0.164
Education	5,440 (81%)	4,281 (82%)	1,159 (78%)	0.003
Alcohol consumption	367 (5.5%)	265 (5.1%)	102 (6.9%)	0.008
Current smoking	1,336 (20%)	1,062 (20%)	274 (19%)	0.142
Waist circumference (m)	0.86 (0.80, 0.92)	0.85 (0.79, 0.91)	0.90 (0.83, 0.97)	<0.001
Systolic blood pressure (mmHg)	137 (126, 150)	136 (125, 149)	144 (132, 153)	<0.001
Diastolic blood pressure (mmHg)	83 (77, 91)	82 (76, 90)	87 (80, 95)	<0.001
Heart rate (bpm)	76 (70, 83)	75 (70, 82)	77 (71, 85)	<0.001
Fasting glucose (mmol/L)	5.40 (5.19, 6.00)	5.40 (5.10, 5.90)	5.70 (5.30, 6.50)	<0.001
Triglycerides (mmol/L)	1.52 (1.14, 2.03)	1.37 (1.06, 1.66)	2.78 (2.29, 3.43)	<0.001
High-density lipoprotein cholesterol (mmol/L)	1.43 (1.23, 1.66)	1.48 (1.30, 1.72)	1.21 (1.07, 1.38)	<0.001
Non-high density lipoprotein cholesterol (mmol/L)	3.31 (2.66, 3.97)	3.18 (2.58, 3.79)	3.81 (3.14, 4.70)	<0.001
Diabetes mellitus	421 (6.3%)	278 (5.3%)	143 (9.7%)	<0.001
Hypertension	1,537 (23%)	1,041 (20%)	496 (34%)	<0.001
Stroke	183 (2.7%)	144 (2.8%)	39 (2.6%)	0.881
Family history of stroke	153 (2.3%)	104 (2.0%)	49 (3.3%)	0.004
CVD high-risk status	1,440 (21%)	985 (19%)	455 (31%)	<0.001
Antihypertensive drug therapy	884 (13%)	581 (11%)	303 (21%)	<0.001
Antidiabetic drug therapy	261 (3.9%)	179 (3.4%)	82 (5.6%)	<0.001
Statin use	188 (2.8%)	122 (2.3%)	66 (4.5%)	<0.001

Continuous variables: median (Q1, Q3). Dichotomous variables: n (%).

Man-Whitney U test; Pearson’s Chi-squared test.

### Association between AIP and CVD high-risk status

In logistic regression, AIP was positively associated with CVD high-risk status. When modeled continuously, each 1-unit increase in AIP was associated with higher odds of CVD high risk in the crude model (OR 2.415, 95% CI 1.888-3.090; *P* < 0.001) and remained significant after full adjustment (Model 3: OR 1.717, 95% CI 1.324-2.227; *P* < 0.001).

Using the categorical definition (cutoff=0.2), participants with high AIP had higher odds of CVD high risk compared with those with low AIP (Model 3: OR 1.553, 95% CI 1.356-1.780; *P* < 0.001) (**Table 2**).

**Table 2:** Associations of AIP with CVD high-risk status.

Variable	Model 1		Model 2		Model 3	
	OR (95% CI)	*P*-value	OR (95% CI)	*P*-value	OR (95% CI)	*P*-value
AIP (per 1-unit increase)	2.415 (1.888, 3.090)	<0.001	2.425 (1.895, 3.103)	<0.001	1.717 (1.324, 2.227)	<0.001
AIP group (High vs Low; cutoff=0.2)	1.882 (1.653, 2.142)	<0.001	1.858 (1.631, 2.117)	<0.001	1.553 (1.356, 1.780)	<0.001

Model 1: crude (exposure only). Model 2: adjusted for alcohol consumption and Married. Model 3: additionally adjusted for waist circumference, education, family history of stroke, antidiabetic drug therapy, antihypertensive drug therapy, and statin use. Binary AIP group uses cutoff=0.2 (High: AIP≥cutoff; Low: AIP<cutoff).

### Dose-response relationship (restricted cubic spline)

**Figure 3:**
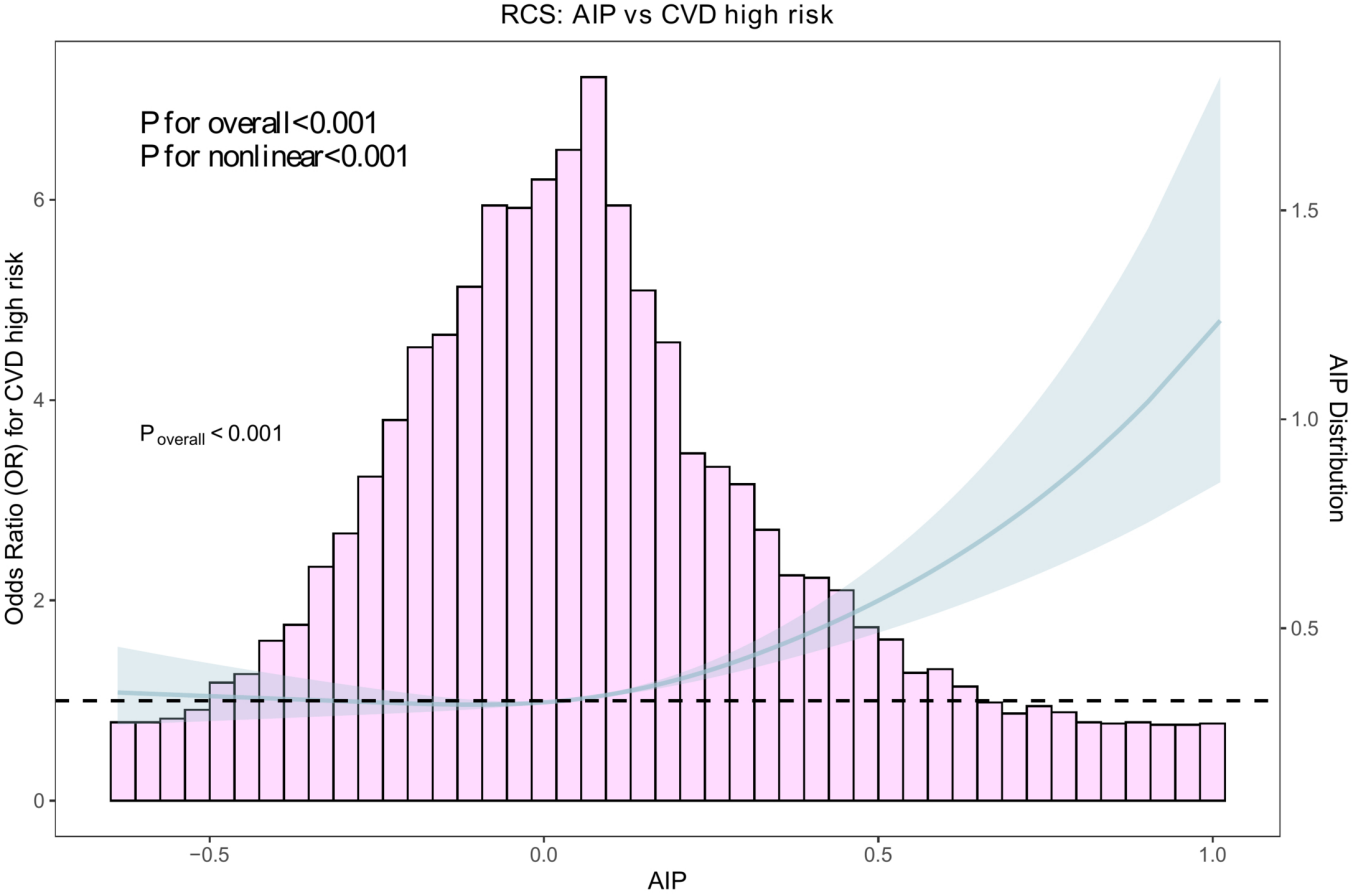
Restricted cubic spline (RCS) analysis of AIP and CVD high-risk status. Restricted cubic spline logistic regression shows the dose-response relationship between AIP and WHO CVD risk chart-defined predicted CVD high-risk status (≥20% vs <20%). Overall and non-linear associations were significant (both *P* < 0.001).

Restricted cubic spline analysis indicated a significant non-linear association between AIP and the odds of CVD high-risk status (*P* for overall <0.001; *P* for nonlinearity <0.001), suggesting that risk increased across the AIP range with a steeper rise at higher AIP levels (**Figure 3**).

### Subgroup analyses

**Figure 4:**
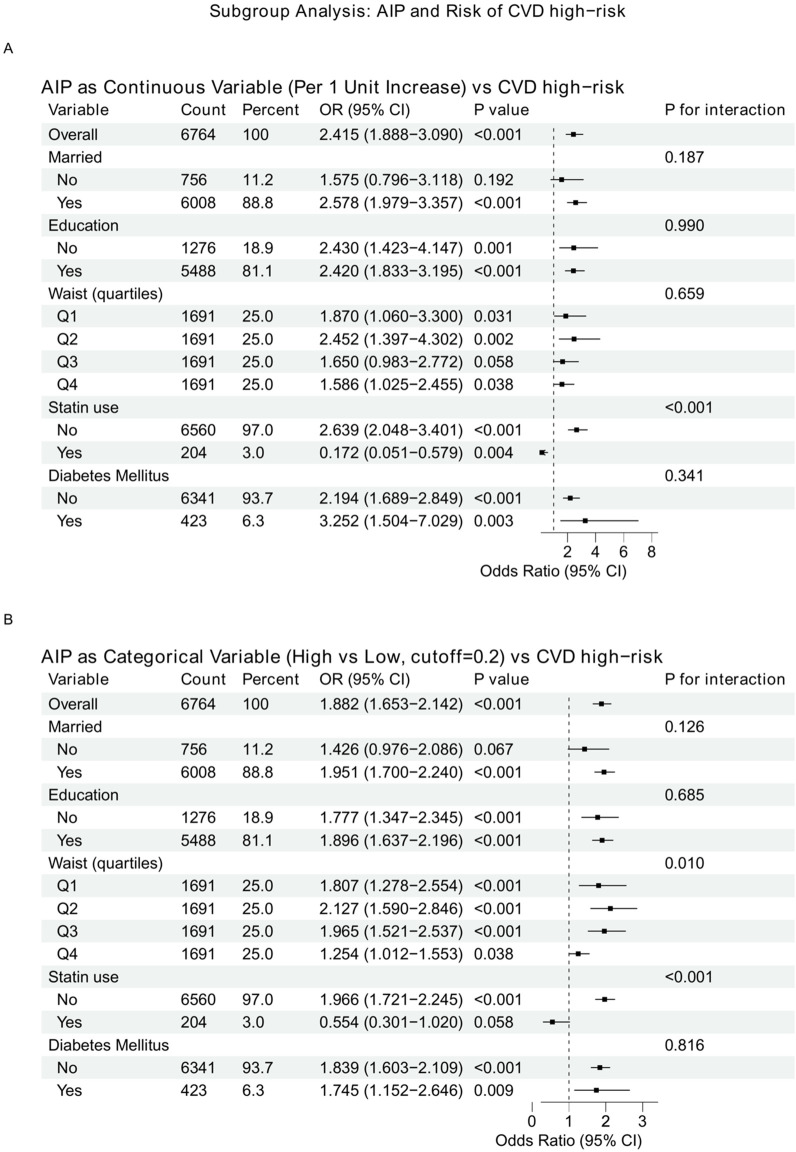
Subgroup analyses of the association between AIP and CVD high-risk status. Forest plots show subgroup-specific ORs (95% CIs) for the association of AIP with CVD high-risk status. **(A)** AIP modeled as a continuous variable (per 1-unit increase). **(B)** AIP modeled as a categorical variable (high vs low, cutoff = 0.2). P for interaction assesses heterogeneity across subgroups.

In subgroup analyses, the direction of association between AIP and CVD high-risk status was generally consistent. Evidence of heterogeneity was observed across waist circumference quartiles (*P* for interaction=0.010) and by statin use (*P* for interaction<0.001), whereas interactions for marital status, education, and diabetes mellitus were not statistically significant. Directionally, the positive association was evident among participants without statin use but was attenuated among statin users in the binary subgroup analysis (**Figure 4**).

### Interaction analyses (predicted probability curves)

**Figure 5:**
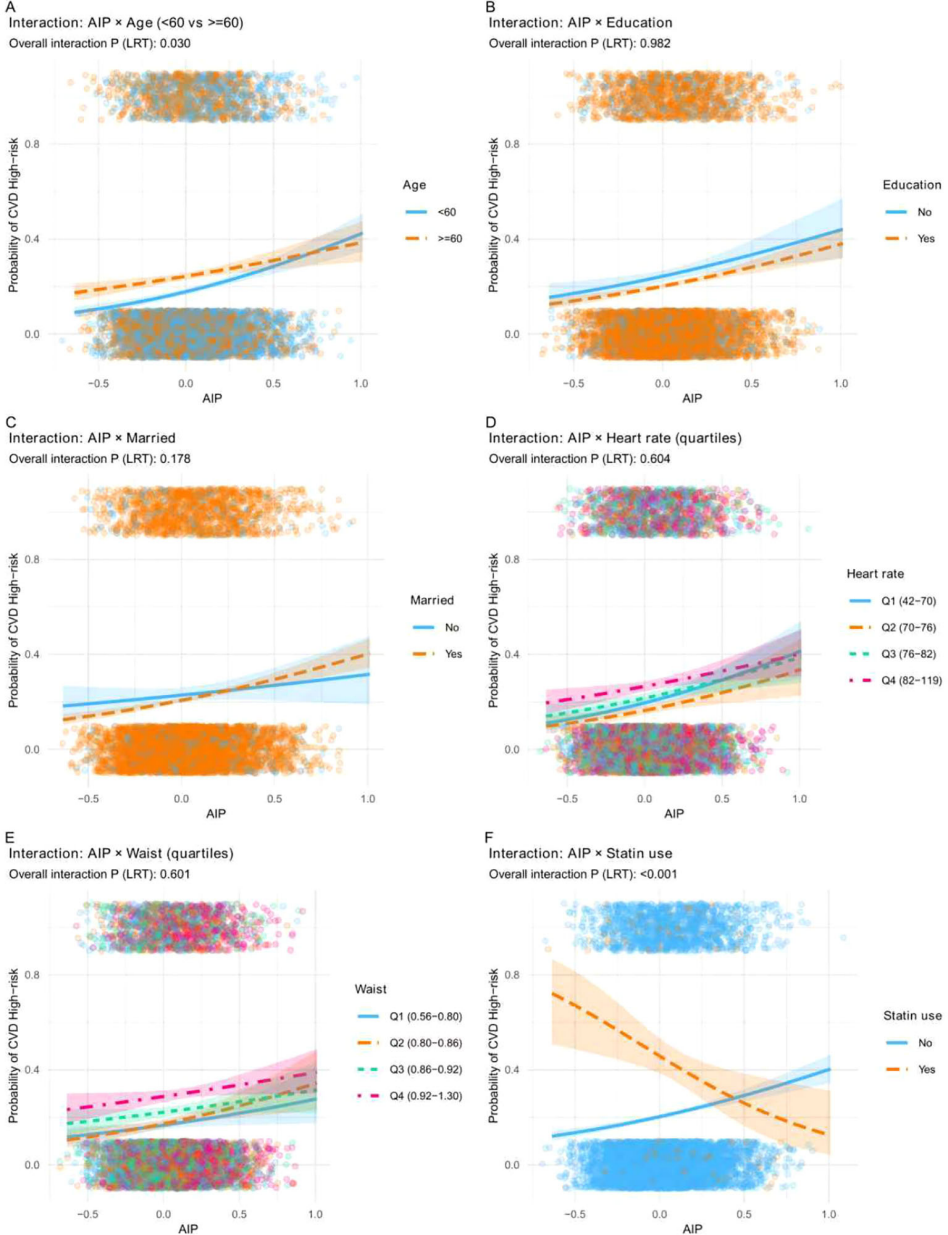
Interaction analyses between AIP and selected factors on the probability of CVD high-risk status. Predicted probability curves illustrate the association between AIP and CVD high-risk status across different strata. **(A)** Age (<60 vs ≥60 years). **(B)** Education (No vs Yes). **(C)** Marital status (No vs Yes). **(D)** Heart rate quartiles. **(E)** Waist circumference quartiles. **(F)** Statin use (No vs Yes). Overall interaction *P* values were derived from likelihood-ratio tests.

In interaction models using continuous AIP, likelihood-ratio tests indicated a significant interaction between AIP and age (<60 vs ≥60 years; overall interaction *P* = 0.030), while the interaction with statin use was also significant (overall interaction *P* < 0.001). In the continuous interaction model, the predicted probability increased with higher AIP among non-users but decreased among statin users. Other tested interactions (education, marital status, heart rate quartiles, and waist quartiles) were not significant (**Figure 5**).

### Explainable machine-learning prediction (LASSO + random forest + SHAP)

**Figure 6:**
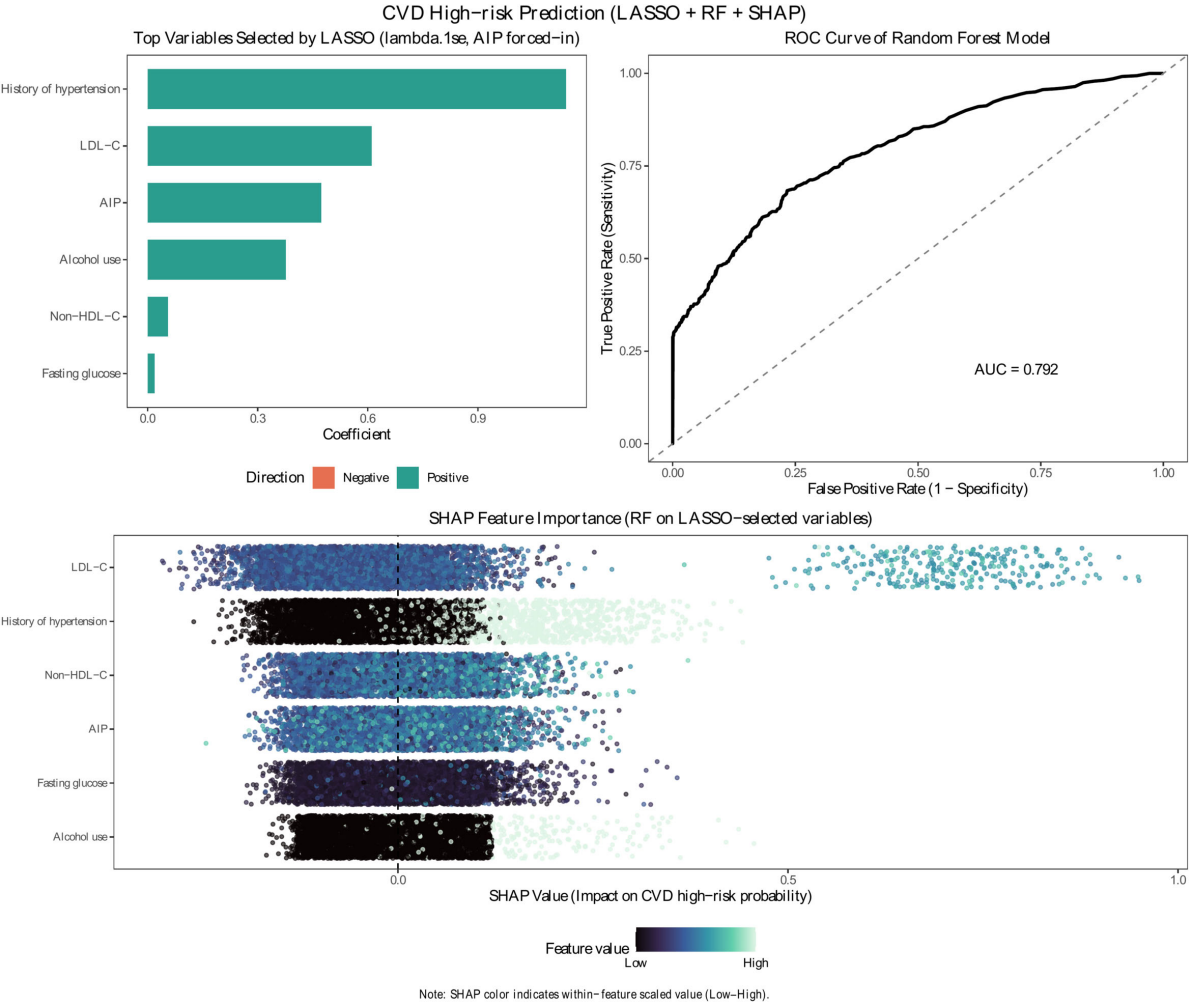
CVD high-risk prediction using LASSO + random forest + SHAP. LASSO (lambda.1se; AIP forced-in) selected a compact set of predictors, which were used to train a random forest classifier (test-set AUC = 0.792). SHAP summary plot shows each feature’s contribution to the predicted probability of CVD high-risk (x-axis: SHAP value; color: within-feature scaled value from low to high). Features are ordered by mean(|SHAP|); thus “rank x of y selected” indicates the x-th ranked feature among the y retained predictors in the final model.

In the explainable machine-learning analysis, LASSO (lambda.1se, with AIP forced-in) identified a compact set of predictors for CVD high-risk classification, including history of hypertension, LDL-C, AIP, alcohol use, non-HDL-C, and fasting glucose. Using these LASSO-selected variables, the random forest model showed good discrimination for CVD high-risk status with an AUC of 0.792 (**Figure 6**). In SHAP reporting, the label “rank x of y selected” denotes that a feature is the x-th most important among the y predictors retained for the final model, where importance is quantified by the mean absolute SHAP value (global feature importance). In our analysis, six predictors were retained (y=6), and the SHAP global-importance ranking was LDL-C (rank 1/6), history of hypertension (rank 2/6), non-HDL-C (rank 3/6), AIP (rank 4/6), fasting glucose (rank 5/6), and alcohol use (rank 6/6) (**Figure 6**). In the supplementary analysis including age and sex, the AUC increased modestly to 0.813 (95% CI 0.790-0.836), and age/sex emerged among the important predictors while AIP remained selected and ranked 5/10 (**Supplementary Figure 2**).

## Discussion

### Principal findings

In this community-based sample (N = 6,702), participants with high AIP (≥0.2) showed a substantially higher prevalence of CVD high-risk status (31% vs 19%). Across logistic regression models, AIP was consistently associated with higher odds of CVD high-risk status; in the fully adjusted model, the odds increased by ~72% per 1-unit increase in AIP (OR 1.717, 95% CI 1.324-2.227), and participants in the high-AIP group had ~55% higher odds than those in the low-AIP group (OR 1.553, 95% CI 1.356-1.780). These findings suggest that AIP, a lipid-derived marker reflecting atherogenic dyslipidemia, captures cardiometabolic risk information aligned with WHO-chart-defined CVD risk categorization (3, 12, 13, 16–20).

### Dose-response pattern and nonlinearity

Restricted cubic spline analysis indicated a statistically significant overall association between AIP and the probability of CVD high-risk, with evidence of nonlinearity (both overall and non-linear components *P* < 0.001), supporting a dose-response relationship that is not strictly linear across the exposure range. This pattern implies that risk increments may vary at different AIP levels, which is clinically relevant when interpreting AIP as a continuous risk marker and when choosing potential thresholds for practical use (16–18).

### Discrimination performance and cutoff determination

Although AIP was associated with CVD high-risk status, its discriminative ability as a standalone marker was modest (AUC 0.557, 95% CI 0.541-0.576), indicating limited classification performance (3, 5, 6). The ROC-derived Youden index suggested an optimal cutoff of 0.231 (95% CI 0.220-0.244), which was used for ROC reporting and sensitivity analysis; in parallel, the rounded cutoff of 0.2 was used for the main baseline comparison and regression/subgroup presentation to improve interpretability. Together, these findings support AIP as a complementary risk indicator rather than a stand-alone screening test, especially when the outcome is defined by multivariable risk charts. In practice, discrimination is expected to improve when AIP is integrated with key clinical determinants in multivariable models, consistent with the higher AUC achieved by our multivariable machine-learning model.

Beyond conventional regression and ROC analyses, the explainable machine-learning pipeline provided a complementary perspective by jointly evaluating multivariable classification and feature importance. Notably, the random forest model built on LASSO-selected predictors achieved an AUC of 0.792, substantially higher than AIP alone, highlighting that CVD high-risk classification is better captured by a combination of lipid-related and clinical factors. SHAP results consistently placed AIP among the top contributing predictors while indicating that LDL-C and history of hypertension carried even larger contributions, which is clinically plausible given their direct links to cardiometabolic risk burden. These findings reinforce the role of AIP as a pragmatic adjunct marker that adds information within a multivariable context rather than functioning as an independent screening test. At the same time, excluding age and sex from the primary ML model may reduce discrimination and makes that model not directly comparable with conventional risk models that include non-modifiable demographic predictors.

### Subgroup patterns and interaction signals

Subgroup analyses generally supported a positive association between AIP and CVD high-risk across strata (e.g., marital status, education, waist quartiles, diabetes status), with formal interaction tests suggesting limited heterogeneity overall but notable effect modification in specific factors. In interaction modeling, the AIP-risk relationship differed by age group (overall interaction *P* = 0.030) and by statin use (overall interaction *P* < 0.001), whereas education, marital status, heart rate quartiles, and waist quartiles did not show strong interaction signals in the plotted interaction models (5–9). The statin interaction may reflect confounding by indication and potential reverse causality, because lipid-lowering therapy can modify TG and HDL-C and thereby shift AIP in this cross-sectional setting. These findings may reflect confounding by indication, lipid modification by treatment, or competing risk-factor burdens across strata, and they underscore that clinical context-particularly statin therapy and age-should be considered when interpreting AIP.

### Potential mechanisms and clinical interpretation

AIP integrates triglycerides and HDL-C, and therefore reflects an atherogenic lipid pattern commonly linked to insulin resistance, increased small dense low-density lipoprotein cholesterol (LDL-C) particles, endothelial dysfunction, oxidative stress, and low-grade inflammation pathways that plausibly contribute to higher predicted CVD risk. The observed non-linear exposure-risk curve further suggests that AIP may capture threshold-like behavior or saturation effects in dyslipidemia-related risk accumulation, which is consistent with the complexity of lipid metabolism and cardiometabolic clustering in middle-aged and older adults (7–13, 25). Recent acute coronary syndrome evidence also suggests that higher AIP is associated with more severe coronary atherosclerosis, including multivessel disease (26).

### Strengths and limitations

Key strengths include a relatively large community-based sample with clear group definitions and internally consistent results across complementary methods (distribution comparison, multivariable regression, spline modeling, ROC/bootstrapping, subgroup and interaction analyses). Limitations should be acknowledged: the cross-sectional design prevents causal inference; residual confounding may remain despite adjustment; some covariates may be subject to measurement or reporting error; and CVD high-risk status is derived from WHO risk charts rather than adjudicated events, which may affect generalizability to “hard” outcomes. In addition, data on statin intensity, duration, and adherence were unavailable, so the observed statin interaction should be interpreted cautiously. Finally, discrimination for AIP alone was modest (AUC 0.557), which is expected because the endpoint is defined by multivariable WHO CVD risk charts rather than by a single biomarker. The machine-learning analysis was exploratory and internally validated only, using a single train-test split, and thus may be subject to optimism and limited generalizability. External validation and repeated resampling (e.g., nested cross-validation) are warranted to confirm the stability of variable importance rankings and predictive performance.

### Implications and future directions

Given its simplicity and reliance on routine lipid measurements, AIP could be useful for rapid risk enrichment in community screening and resource-limited settings, particularly as a complementary indicator alongside established multivariable tools such as WHO CVD risk charts. Future studies should validate AIP thresholds in independent cohorts, compare AIP with other adiposity/lipid-derived indices under the same outcome framework, and-most importantly-assess whether AIP predicts prospective CVD events beyond traditional risk factors and whether therapy status (e.g., statin use) systematically modifies its prognostic meaning (1, 3, 4).

## Conclusion

In this community-based ChinaHEART population, AIP was positively and non-linearly associated with WHO CVD risk chart-defined predicted CVD high-risk category (rather than adjudicated or incident CVD events) after multivariable adjustment. As a standalone marker, AIP showed limited discriminatory performance (AUC ≈0.56), and the ROC-derived threshold (~0.23; rounded to 0.2) should be considered exploratory rather than a screening cut-point. Notably, in an explainable machine-learning framework, AIP remained among the top predictive features alongside LDL-C and history of hypertension, supporting its role as a low-cost adjunct to prioritize individuals for more comprehensive risk assessment in primary-care screening settings. Prospective studies with external validation are warranted to confirm clinical utility and optimal thresholds for predicting incident CVD events.

## Data Availability

The raw data supporting the conclusions of this article will be made available by the authors, without undue reservation.

## Supplementary material

Supplementary Figure 1 Sensitivity subgroup analysis of the association between binary AIP and CVD high-risk status using the original cutoff. Forest plot shows subgroup-specific ORs (95% CIs) for high versus low AIP, defined using the original ROC/Youden-derived cutoff of 0.231. The overall direction of association remained similar to that in the main analysis. *P* for interaction assesses heterogeneity across subgroups.

Supplementary Figure 2 Supplementary machine-learning sensitivity analysis including age and sex. The left panel shows the ROC curve of the random forest sensitivity model including age and sex, with a test-set AUC of 0.813 (95% CI 0.790–0.836). The right panel shows the corresponding SHAP summary plot, illustrating each feature’s contribution to the probability of CVD high-risk; features are ordered by mean absolute SHAP value. In this supplementary analysis, age and sex emerged among the important predictors, while AIP remained selected.
