# Supplementary figures and images for: Atherogenic index of plasma and cardiovascular high-risk status in the ChinaHEART luohe cohort: multivariable association modeling with nonlinear dose-response and effect heterogeneity
Source: Front Endocrinol (Lausanne). 2026 Apr 1;17:1798735. doi: 10.3389/fendo.2026.1798735 (PMC13079028; doi:10.3389/fendo.2026.1798735)

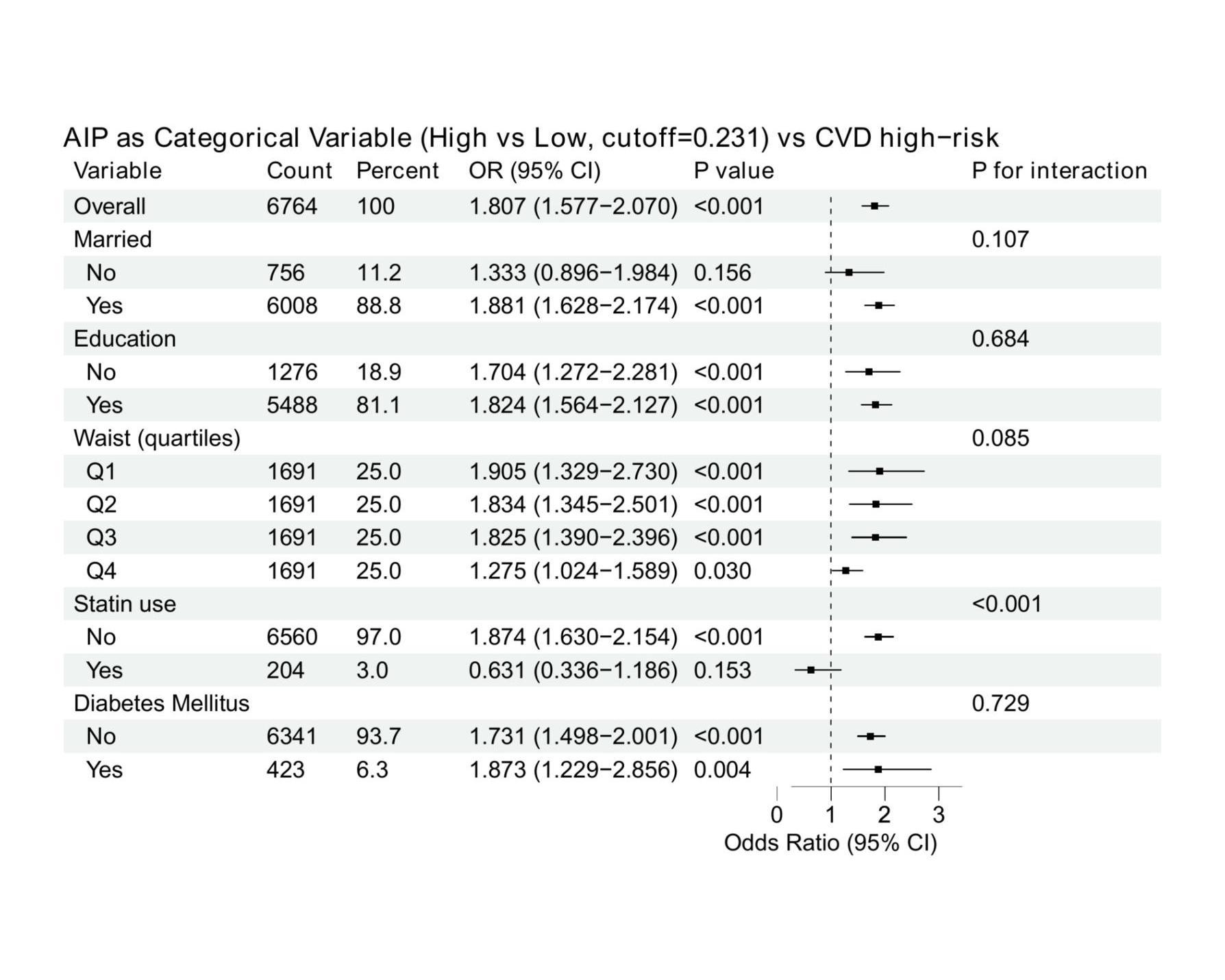

Supplement: Supplementary Figure 1 — Sensitivity subgroup analysis of the association between binary AIP and CVD high-risk status using the original cutoff. Forest plot shows subgroup-specific ORs (95% CIs) for high versus low AIP, defined using the original ROC/Youden-derived cutoff of 0.231. The overall direction of association remained similar to that in the main analysis. P for interaction assesses heterogeneity across subgroups. [file Image1.jpeg]

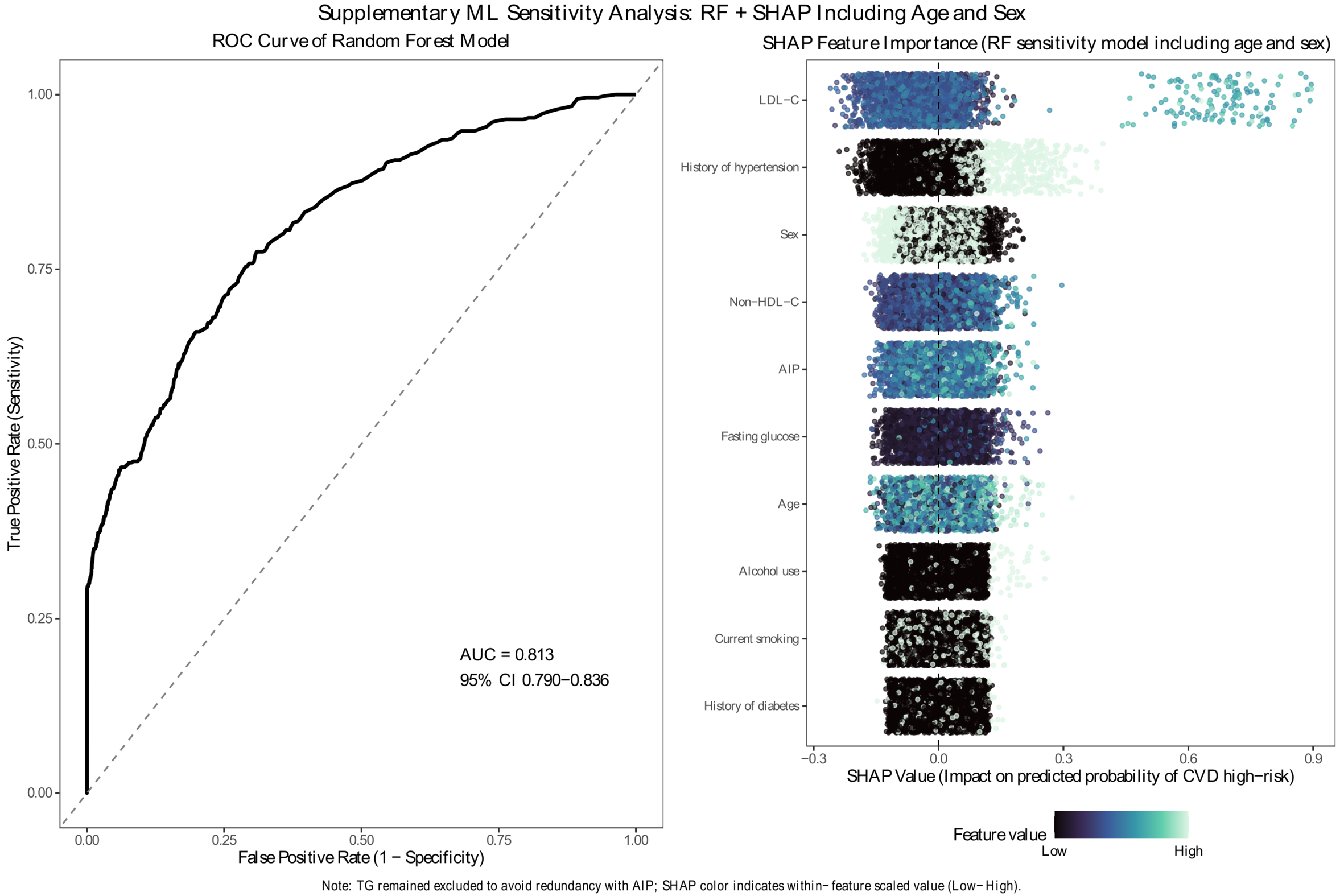

Supplement: Supplementary Figure 2 — Supplementary machine-learning sensitivity analysis including age and sex. The left panel shows the ROC curve of the random forest sensitivity model including age and sex, with a test-set AUC of 0.813 (95% CI 0.790–0.836). The right panel shows the corresponding SHAP summary plot, illustrating each feature’s contribution to the probability of CVD high-risk; features are ordered by mean absolute SHAP value. In this supplementary analysis, age and sex emerged among the important predictors, while AIP remained selected. [file Image2.jpeg]
